# The horizon of pediatric cardiac critical care

**DOI:** 10.3389/fped.2022.863868

**Published:** 2022-09-16

**Authors:** Uri Pollak, Yael Feinstein, Candace N. Mannarino, Mary E. McBride, Malaika Mendonca, Eitan Keizman, David Mishaly, Grace van Leeuwen, Peter P. Roeleveld, Lena Koers, Darren Klugman

**Affiliations:** ^1^Section of Pediatric Critical Care, Hadassah University Medical Center, Jerusalem, Israel; ^2^Faculty of Medicine, the Hebrew University of Jerusalem, Jerusalem, Israel; ^3^Pediatric Intensive Care Unit, Soroka University Medical Center, Be'er Sheva, Israel; ^4^Faculty of Health Sciences, Ben-Gurion University of the Negev, Be'er Sheva, Israel; ^5^Divisions of Cardiology and Critical Care Medicine, Department of Pediatrics, Northwestern University Feinberg School of Medicine, Ann & Robert H Lurie Children's Hospital of Chicago, Chicago, IL, United States; ^6^Divisions of Cardiology and Critical Care Medicine, Departments of Pediatrics and Medical Education, Northwestern University Feinberg School of Medicine, Ann & Robert H Lurie Children's Hospital of Chicago, Chicago, IL, United States; ^7^Pediatric Intensive Care Unit, Children's Hospital, Inselspital, Bern University Hospital, Bern, Switzerland; ^8^Department of Cardiac Surgery, The Leviev Cardiothoracic and Vascular Center, The Chaim Sheba Medical Center, Tel Hashomer, Israel; ^9^Pediatric and Congenital Cardiac Surgery, Edmond J. Safra International Congenital Heart Center, The Chaim Sheba Medical Center, The Edmond and Lily Safra Children's Hospital, Tel Hashomer, Israel; ^10^Pediatric Cardiac Intensive Care Unit, Sidra Medicine, Ar-Rayyan, Qatar; ^11^Department of Pediatrics, Weill Cornell Medicine, Ar-Rayyan, Qatar; ^12^Department of Pediatric Intensive Care, Leiden University Medical Center, Leiden, Netherlands; ^13^Pediatrics Cardiac Critical Care Unit, Blalock-Taussig-Thomas Pediatric and Congenital Heart Center, Johns Hopkins Medicine, Baltimore, MD, United States

**Keywords:** training, personalized medicine, artificial intelligence, tissue engineering, safety and quality, pediatric cardiac critical care, minimally invasive cardiac surgery, mechanical circulatory support

## Abstract

Pediatric Cardiac Critical Care (PCCC) is a challenging discipline where decisions require a high degree of preparation and clinical expertise. In the modern era, outcomes of neonates and children with congenital heart defects have dramatically improved, largely by transformative technologies and an expanding collection of pharmacotherapies. Exponential advances in science and technology are occurring at a breathtaking rate, and applying these advances to the PCCC patient is essential to further advancing the science and practice of the field. In this article, we identified and elaborate on seven key elements within the PCCC that will pave the way for the future.

## Introduction

In 1671, Neils Stenson described the cardiac pathology of a stillborn fetus with multiple congenital anomalies including the cardiac lesion, which is now recognized as tetralogy of Fallot (1). The first palliative intervention for these patients was pioneered by Hellen Taussig and Alfred Blalock, in November 1944, with the assistance of Vivian Thomas, when the left subclavian artery was anastomosed to the pulmonary artery, with what now known as the Blalock-Thomas-Taussig shunt, in a severely cyanosed child with tetralogy of Fallot (2). A decade later, Sir Walter Lillehei performed the first complete repair for patients with tetralogy of Fallot using human cross-circulation technique (3). During these procedures, children were hand-ventilated and endotracheal tubes were removed on the table at the end of the procedure (4). Following extubation, postoperative care proceeded in the hospital ward, where children were placed in a closed oxygen tent. No invasive monitoring or arterial blood gases were undertaken, and all medications such as morphine and penicillin, were administered by intramuscular injections (4).

**Figure 1:**
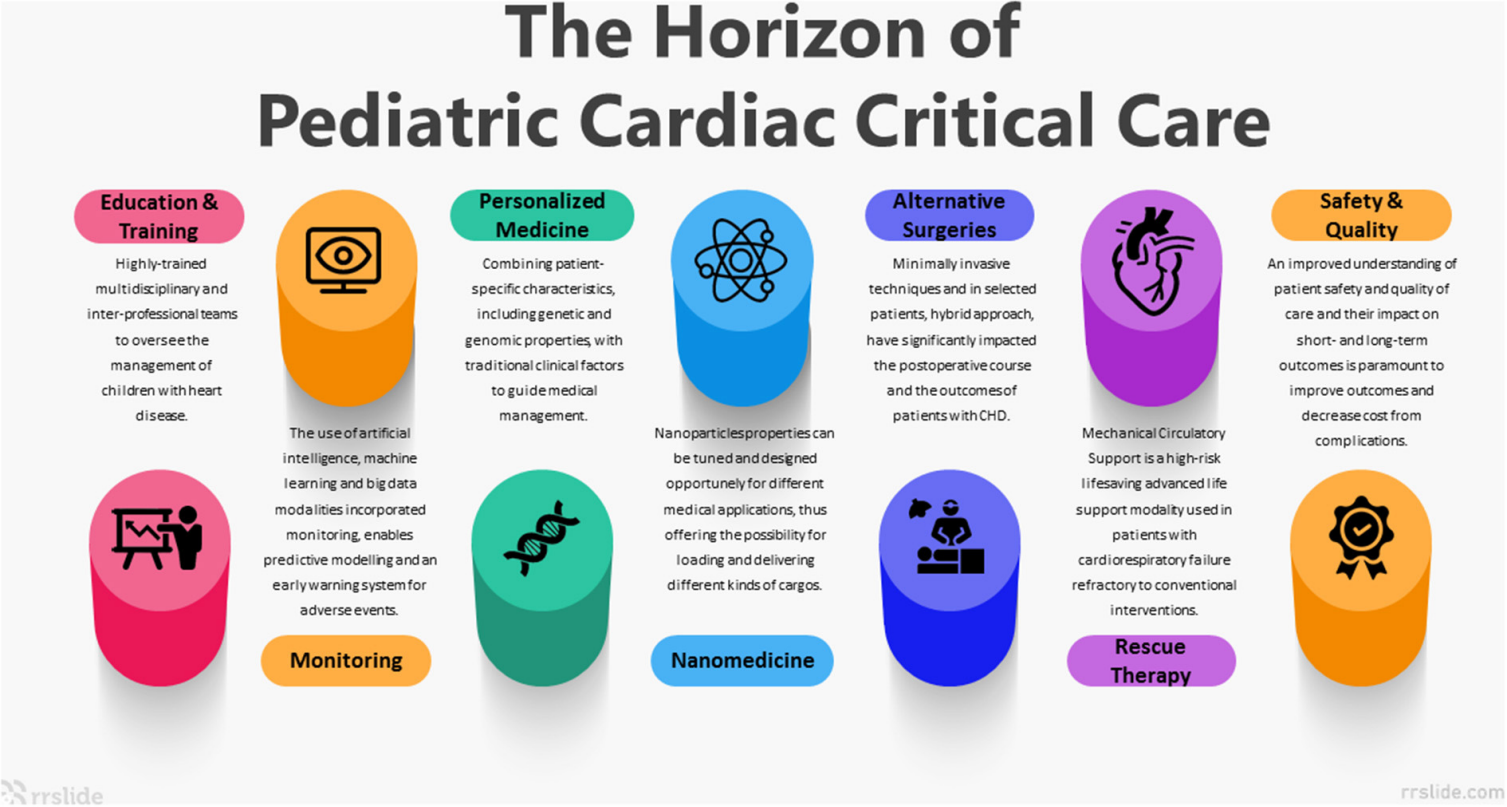
Seven Pediatric Cardiac Critical Care key elements that will pave the way to the coming decades.

The field of pediatric cardiac critical care (PCCC) has developed rapidly in the last 30 years. In the modern era, the partnership between congenital cardiac surgery and PCCC has resulted in dramatically improved outcomes, driven largely by transformative technologies and an expanding collection of novel pharmacotherapies. The exponential advances in science and technology are occurring at a breathtaking rate; applying these advances to the PCCC patient will be essential to advancing the science and practice of the field. In this article, we identified and elaborate on seven key elements within the PCCC that will pave the way to the coming decades (Figure 1).

## Future perspectives of pediatric cardiac critical care

### Education and training

In response to PCCC growing complexity, more hospitals are using dedicated pediatric cardiac units with highly trained multidisciplinary and inter-professional teams to oversee the management of children with heart disease (5, 6). This care model requires specialized training to provide high-quality care, keep up with evolving technologies, and to perform with optimal teamwork and communication in a complex environment (6–8).

In the circles of medical education, we recognize the need for standardized instruction and assessment so that learners are achieving and maintaining skills, knowledge and attitudes to ensure our highest standards of care. Competency-based medical education (CBME) has become the primary strategy in the United States (US) and other countries to provide a standardized education and assessment of trainees with the use of milestones and entrustable professional activities (EPAs) (9). Milestones, developed and implemented by the Accreditation Council for Graduate Medical Education (ACGME; organization that defines standards for US residency and fellowship programs), is a competency-based assessment tool used to help standardize the trainee experience (10). Milestones of trainees are assessed bi-annually in the domains of medical knowledge, patient care, professionalism, and system-based practice (11). EPAs are defined as measurable activities delineated by each discipline to observe that a trainee can perform a given task independently (12). Learners can then be assessed for each task, and with the provision of effective feedback, the learner builds graduated responsibility and competency in specific educational domains (9, 12).

Learning objectives offer further specificity, and these are noted for PCCC in the physician curriculum published by the Pediatric Cardiac Intensive Care Society (PCICS), as well as a recent publication by Tabbutt et al. (13, 14). Standardization and structured educational opportunities for nurses and advanced practice providers are also expanding; PCICS also recently published an advanced practice provider and a nursing curriculum to further the effort to standardize knowledge and skills for those disciplines (15–17).

Simulation-based training is a frequently used educational modality for trainees and staff in all levels and in different disciplines (18). There is growing evidence that simulation-based education strategies, including mastery learning curriculum can improve patient care practices as well as improved outcomes (19–21). Using principles of adult learning theory with methodology in simulation, the Simzones framework, was developed as a graduated learning system to develop simulation-based activities for adult learners, although the integration of mutli-modal simulation technologies has not been well explored (22–25). In the US, simulation curricula have been developed for first year pediatric critical care fellows and cardiology fellows respectively (26–28). PCCC simulation has focused on building understanding of physiology as well as multidisciplinary teamwork and systems of care through bootcamps and other simulation sessions (23). Multi-center creation of simulation scenarios may further help standardize learner training from different disciplines and institutions (13, 14).

Virtual reality (VR) has increasingly been utilized as an adjunctive educational tool in medical education training, particularly in non-technical skill building including communication and team building situations (29). VR-based training may provide improved accessibility by using a computer or virtual platform, allowing trainees to learn asynchronously and independently (30, 31). The use of VR simulations for junctional ectopic tachycardia and low cardiac output syndrome have been used for physician trainees, noting that participants had positive feedback using this interface (32). The Stanford Virtual Heart project has been developed as another resource for learners to interact with various congenital heart malformations, focus on their spatial relationships with other heart structures (31). Further research is needed to describe the effectiveness of learning with this new technology, including in PCCC (32).

Three-dimensional (3-D) printing models are another educational modality for multidisciplinary learners' use (33–35). Hussein et al. developed a 3-D printed heart model for surgical trainee practice for the arterial switch operation and demonstrated an improvement of time and trainee technical performance when using 3-D printed model before using a hands-on training congenital heart surgery tool (36). 3-D printing requires special software and technology and is currently only available at specific institutions. Further research is needed to demonstrate improved educational effects and subsequent performance on patient outcomes.

### Patient monitoring

Daily work in the PCCC unit involves multitasking, often with disparate teams and unpredictable high-risk events. These factors combine to produce an environment in which health providers must rely on knowledge and protocols/warning systems to guide the prioritization and performance of tasks (37). Due to the significant presence of and reliance on technology in such modern healthcare environments, the interaction between healthcare providers and these technologies may have a profound impact on the quality of care (37). In order to improve outcomes, significant effort has been dedicated to designing tools to help ICU providers manage the increasing influx of data, and facilitate the early identification of patient deterioration and risks.

Development of such algorithms has continued, and they now cover multiple data streams and are designed as risk assessment tools. An example of newer generation algorithms is the Rothman Index (RI), developed for adults, which is an illness-severity index embedded within the electronic medical record. Twenty-six variables are continuously tracked, and data is fed into a proprietary algorithm; the calculated score is designed to reflect patient illness severity (37).

Another pediatric prediction tool assesses imbalances between oxygen delivery and oxygen consumption, associated with organ dysfunction along with morbidity and mortality. The Inadequate Oxygen Delivery (IDO_2_) Index (Etiometry, Boston MA) synthesizes patient physiologic and laboratory measures to continuously predict the risk of having a mixed venous oxygen saturation < 40%, wherein an elevated IDO2 value indicates elevated risk in children following CPB surgery (38). This web-based tool captures and displays integrated data exported from continuous bedside physiologic data.

Lin et al. investigated the usability of data integration and visualization of T3 in the light of human factors and discovered several limitations to the easy implementation of the software (39). The observations from usability testing warn that without consistent exposure and integration into clinical practice, data interpretation aids may be ignored, and, thus, excluded from critical decision-making where they would be most useful. Furthermore, a study comparing low cardiac output score and IDO_2_ for predicting adverse events in 72 h following congenital heart surgery showed that using the IDO_2_ values had no association with occurrences of adverse events (40). A group from Boston Children's found that IDO_2_ monitoring could identify critically ill children with sepsis at highest risk of adverse events or undesirable outcomes (41), and Dewan et al validated the IDO_2_ index (IDO_2_) to predict in-hospital cardiac arrest in a general pediatric ICU (42). Additional specific congenital heart disease machine-learning (ML) approaches to identify risk factors for complications in the early postoperative phase (43), long-term complications (44), prediction of brain injuries in ECMO patients (45) are published as well. These ML algorithms were used to predict clinical deterioration, to classify surgical risk, or to classify the heart disease using patient characteristics. Early prediction of critical events in infants using a naïve Bayesian model was introduced by Ruiz et al. Thirty-four routinely collected data points, such as heart rate, CO2, and lactate, were integrated into the algorithm. The model was able to predict events up to 1 h prior to their occurrence with high sensitivity and specificity (46). Single ventricle lesions remain high risk for adverse events. A novel ECG algorithm utilizing ST segment instability for detection of cardiopulmonary arrest in single ventricle physiology was described by Vu et al (47).

Results of recent studies on AI algorithms in patients with CHD are encouraging. Nevertheless, patient monitoring algorithms remain in an early phase, and ongoing development is likely in the following realms: (I) scope of input (II). algorithm parameters, (III) human-machine interfaces, and (IV) training (how to use such indicators as decision support tools) (48). While perhaps promising, the costs of implementation are very high and the prospect of being universally available is not likely in the near future. These applications should only supplement standard monitoring but not substitute for current standards of ICU monitoring.

### Genomic medicine

Personalized medicine refers to combining patient-specific genetic and genomic properties with traditional clinical factors to guide medical management. The use of the patient-specific data facilitates personalized management, tailored to address individual risk factors, severity of illness and assess response to treatment (49).

PCCC medicine will likely benefit significantly from increased integration and application of genomic medicine. The widely used molecular studies diagnose only about 20% of suspected genetic diseases, and in the Congenital Heart Disease Genetic Network Study designed by the Pediatric Cardiac Genomics Consortium, only 11% of cases had a genetic diagnosis (50). Technologies such as rapid whole genome sequencing (rWGS) of patients admitted in the PCCC units increase the rate of diagnosis and may reduce the cost of care (51). Genomics research also focuses on understanding and treating acquired diseases such as distinguishing viral and bacterial infections in cases of fever (52–54) and may support the decision to perform a semi-elective procedure in a febrile child. Additionally, genomics may also assist in assessing the severity of disease and likelihood of morbidity and mortality in various pathologies, particularly in patients with multiple genetic anomalies and comorbidities. For instance, fatal acute myocarditis has been previously shown to correlate with putatively damaging variants in genes related to cardiomyocyte structure and function (55).

The potential of multi-omics technologies to elucidate the complex interactions between genes, proteins, and biochemical reactions can hopefully fill gaps in our current PCCC knowledge. It can also provide accurate and rapid data to direct management considering the advances in technology and statistical processing power achieved during the last decade.

To date, genomic research provides a better understanding of why some patients develop critical care syndromes such as acute respiratory distress syndrome (ARDS), acute kidney injury (AKI), or severe sepsis, whereas others do not (56, 57). These findings can be applied to the PCCC practices in several aspects. AKI is a common complication of post CPB patients and prediction models of high-risk patients using genomics will potentially modulate their postoperative course. Studies using genome-wide RNA transcriptome analyses of blood enable us to identify groups of children at high risk of mortality and differentiate those likely to benefit from early corticosteroid treatment (58–61). Weathington et al. focused on cell gene expression in severe asthma, revealing mechanisms of severe disease as well as the influences of medications and the identification of severity-related genes which may provide new diagnostic and therapeutic targets (62). Werner et al. reported a detectable signal in gene expression profiles for early detection of ventilator-associated pneumonia in ventilated children (63), a method that may influence the duration of mechanical ventilation in the post cardiac surgery pediatric patient.

Pharmacogenomic research investigates the patient's genetic information that influences their response to therapeutic drugs. One example is the use of pharmacogenomics to adjust sedation and analgesia in pediatric ARDS. Zuppa et al. revealed several factors affecting the pharmacokinetics of midazolam in children, using the Illumina HumanOmniExpress genome-wide single nucleotide polymorphism chip. These findings provide the basis for future implementation of a personalized approach to sedation (64, 65).

Cardiac critical care in general and specifically in the pediatric population provides broad operational leeway for genomics, where a genetic basis was found for an increasing number of congenital defects and pathologies once considered idiopathic (e.g., pulmonary arterial hypertension, Hypoplastic Left Heart Syndrome, and dilated cardiomyopathy) (66–70). Multigene next-generation sequencing panels that focus on cardiomyopathy- or arrhythmia-disease genes are available, and Ritter et al. reported that the results influenced the medical decision-making in 53% of all cases and up to 80% of cases with a positive result, especially when testing was expedited (71).

Most patients in PCCC units are admitted after cardiothoracic surgeries for congenital and acquired heart defects. Reed et al. reported a proteomic analysis of infants who underwent open heart surgery. Some patients experience a systemic response to CPB with significant derangements in hemostasis and systemic inflammation that cause excess morbidity and mortality. This multifactorial response involves acute phase response, coagulation, and cell signaling pathways that are not fully understood yet. Reed et al. identified several biomarkers that improve our understanding of the phenomenon (72). Future implications include early identification and treatment of susceptible infants, likely leading to improved outcomes. However, more research is needed to enable these findings to become feasible tools for intensivists.

Despite the advances made in the field of orthotopic heart transplantation (OHT), there are still gaps in the understanding of the alloimmune response, the role of immunomodulation, development of tolerance, and xenotransplantation. Ongoing research is focused on improving outcomes post OHT using immunomodulation for children with pre-formed anti-HLA antibodies (PRA). Previous studies reported these children to have increased risk for rejection, coronary artery vasculopathy and mortality (73–75). Nowadays, with the increased incidence of Ventricular Assist Device (VAD) therapy which is associated with the development of PRA, improving immunomodulation is crucial as immunomodulation therapies showed better outcomes for PRA-positive children after OHT (76). Genomic research achieved advances in understanding the role of regulatory T cells, costimulatory signals and exosomes, all of which have clinical implications and may be leading targets to promote cardiac allograft tolerance and enable cardiac xenograft survival (77).

While genomic research is surging, ethical and translation challenges arise as well. There is a need to create a suitable model for incorporating genomic data in critical care management. Challenges include knowledge gaps among intensivists on how to interpret genetic results, concerns regarding the potential effect of genetic information on child-parent bonding, and the implications of such information on medical and family decisions (78, 79). Dimmock et al. reported that clinicians perceived rapid genomic sequencing (RGS) to be helpful in 77% of cases and that RGS changed clinical management in 28% cases. Clinicians also reported a low likelihood of harm of RGS of infants in ICUs with diseases of unknown etiology (80). This perception is supported by parents' responses in a study by Cakici et al., describing that most parents reported they had been adequately informed to consent, understood the genetic results, and denied having regrets or experiencing harm from the sequencing (81).

### Regeneration, nanotechnology and tissue engineering

#### Regeneration of cardiomyocytes

Myocardial damage has been traditionally managed with medication or assist devices, depending on the etiology, extent and presentation of the dysfunction. With an estimated turnover rate of <1% per year, with most renewal events reported to occur in the first decade of life, revealing the heart's capacity for regeneration, and how to regulate it, are fundamentals to cardiovascular research (82).

Cardiomyocyte (CM) necrosis, as seen after myocardial infarction (MI) triggers a marked inflammatory response orchestrated mainly by cardiac fibroblasts, and the idea of converting a portion of these cells *in situ* to contractile cells is a transformative concept. Combinations of specific epigenetic modulators or pharmacological inhibition of signaling pathways can improve the conversion of fibroblasts to induced cardiomyocyte-like cells (iCMs). In the initial studies, using viral vectors loaded with cardiogenic transcription factors injected directly into the necrotic area, a modest proportion of CMs in the necrosis border zone was traced as progeny of infected fibroblasts, concomitant with reduced scar area and improved myocardial function (83). Although the robustness of the *in vivo* reprogramming process and the use of viral vectors are under debate, this technique provides a novel, cell-free platform for cardiac repair.

Another important element in the regeneration of CMs is the extracellular matrix (ECM). Extracellular biomechanical properties, such as matrix rigidity, that affect cytoskeletal integrity and sarcomere organization in CMs might act within signaling pathways to influence proliferation (e.g., the Hippo signaling cascade with its transcriptional coactivators YAP and TAZ) (84, 85). The link between Hippo signaling and the sarcomere was further elucidated by Bassat et al. (86) and Morikawa et al. (87) reporting that the dystrophin glycoprotein complex (DGC) inhibits YAP nuclear localization by sensing mechanical and biochemical inputs from the ECM. Agrin, a matrix glycoprotein, promotes CMs cell division *in vitro via* the DGC-YAP axis and is required for an effective regenerative response in the myocardium of neonatal mice. Administration of agrin facilitates cardiac regeneration in adult mice after MI. CMs division might also be modulated by emergence at birth from somewhat hypoxic environment *in utero* to atmospheric oxygen. Recent studies have reported proliferative effects of experimental hypoxia on CMs *in vivo*, making regulated hypoxia worthy of further exploration in the context of the regenerative response (88). The potential benefits of regeneration of damaged cardiac tissue after the direct effect of open heart surgery and the indirect effect of CPB, although still premature and futuristic, are promising and may alter the post-operative course in the PCCC unit.

#### Nanotechnology

Nanomedicine is the application of nanotechnology to medicine for diagnosis and therapy (89). Introducing nanoparticles (NPs) directing modulators of developmental pathways in CMs significantly advanced the concept of cell-level *in-vivo* cardiotherapy. Currently, nanoparticles properties can be tuned and designed for different medical applications, thereby offering the possibility for loading and delivering a multitude of therapies. Special attention is being paid to NP-based system for cardiotherapy and their therapeutic cargos such as microRNAs, cardioprotective drugs or growth factors. The Hippo pathway is a promising target for nanoparticle based therapies (90), as it has emerged as a possible switch in CMs proliferation (91), being tightly connected to the onset and progression of cardiomyopathies (92).

In this context, Nguyen et al. (93) used matrix metalloproteinase (MMP)-responsive hydrogels with the ability to be retained at the necrotic area thus being potentially suitable for the sustained delivery of therapeutic molecules. Another recently reported strategy called *THEREPI* relies on the use of a biocompatible patch, which is placed on the epicardium at the border zone of the necrotic myocardial tissue to achieve the sustained delivery of drugs, macromolecules and possibly cells for cardiac therapy (94). Ideally, *THEREPI* can be efficiently used for the *in situ* administration of therapeutic nanoparticles, thereby increasing their retention at the diseased site and improving cargo delivery.

The potential of gene editing in the restoration of contractility along with the discovery that the clustered regularly interspaced short palindromic repeats (CRISPR)/CRISPR-associated (Cas) system could be used to introduce sequence-specific DNA cleavage in human cells has revolutionized research (95). Nanomedicine-related sciences and different systems have been engineered for carrying the CRISPR/Cas9 apparatus and guide the genetic reprogramming inside the cells, based on lipid and inorganic nanoparticles (96, 97). While the exploitation of this technique in nanomedicine in the context of myocardial regeneration can be attractive in the case of genetically-determined cardiac pathologies, technical challenges connected to its specificity are still under investigation (98). A promising strategy to overcome these challenges involves utilizing organ-on-a-chip technologies, capitalizing on microfluidic advances which are combined with complex three-dimensional (3D) cell biology that provides organ-like physiology and pathophysiological cellular and tissue level responses (99, 100).

#### Tissue engineering

Current surgical procedures used in CHD are limited by the use of prosthetic materials used to replace heart valves, vascular grafts, and synthetic patches. Use of these materials is susceptible to complications such as infection, host immune response, and thrombotic complications. The lack of growth and remodeling potential is also a prominent limitation in children. The field of tissue engineering holds promise for surgical solutions for these patients (101). Tissue engineering, first described as a field by Langer and Vacanti in 1993, promotes using the body's natural growth and regeneration processes to repair and replace damaged and nonfunctioning organs with healthy, native tissue (102). Many approaches exist within tissue engineering, including the use of biodegradable polymeric scaffolds, decellularized extracellular matrix, stem cells, and harvested patient cells (103). The field of congenital cardiovascular tissue engineering that has advanced furthest to date is the tissue engineered vascular grafts such as cavo-pulmonary conduits during the final stage of the Fontan procedure, which express healing, remodeling and growth characteristics of native tissue (101).

Tissue engineered heart valves (TEHVs) remain another challenging field. *In vivo* and preclinical studies have been promising, but clinical translation requires improved performance of current prosthetic options (104). TEHVs have had a difficult history in the clinic, being used in patients after only limited animal models, and being limited by several complications in early studies. These difficulties led to a return to laboratory research to improve the designs, and mechanistic studies of tissue formation in TEHVs are required for further advancement (105).

Many congenital cardiac anomalies can be discovered and diagnosed *in utero* during routine physician appointments (106). If dysfunctional valve associated CHDs could be repaired *in utero* (e.g., balloon valvuloplasty in the fetus to open stenotic valves) there is the potential to provide curative treatment for CHD before birth, preventing the need for any surgeries (107). Combination of this idea of fetal intervention, tissue engineering and scar-free wound healing properties, holds potential to develop novel curative procedures for CHDs (108).

Finally, a group from Tel Aviv University reported on the development and application of advanced 3D printing techniques using the personalized hydrogel as a bioink. Combined with the patient own cells, the hydrogel may be used to print thick, vascularized, and perfusable cardiac patches that fully match the immunological, biochemical and anatomical properties of the patient. The personalized hydrogel was used to print volumetric, freestanding, cellular structures, including whole hearts with their major blood vessels (109).

As we look toward the horizon, novel technologies developed through nano-medicine and tissue engineering, can be expected to change the patient care in the PCCC. It will be important for pediatric cardiologists, cardiac intensivists and cardiac surgeons to accelerate this research and ensure that the new technologies are applied toward the treatment of the critically ill pediatric cardiac patient.

### Alternatives to the traditional cardiac surgery

#### Minimally invasive cardiac surgery

During the last decades there has been tremendous advancement in minimally invasive techniques for most of the surgical fields, including congenital heart defects (CHD). While only extracardiac defects, such as PDA, were correctable during the first years of minimally invasive approach, advancements in the field now allow some complex intracardiac defects to also be repaired or palliated with a minimally invasive approach (110).

The use of these techniques significantly impacts the postoperative course and the outcomes of patients with CHD. First, it allows earlier mobility and resumption of physical activity secondary to reduced pain and respiratory dysfunction and thereby shortens the length of hospital stay. Furthermore, it reduces the long-term morbidity related to sternotomy, such as chest wall asymmetry, rib fusion, scoliosis, shoulder girdle abnormalities, chronic pain syndrome and more. Lastly, it is associated with a number of cosmetics benefits (110, 111). Complete care of children with CHD does not concise of merely repairing or palliating their heart defect, but rather ensuring their psychosocial future wellbeing. A better cosmetic result has been associated with an improved self-body image and quality of psychosocial wellbeing.

Various approaches exist for minimally invasive pediatric cardiac surgery. The choice depends on the anomaly type and surgical preferences. Extracardiac malformations such as PDA, vascular ring, aortic coarctation, collateral vascular system closure and ligation and more, can be performed *via* left lateral thoracotomy, but also using video-assisted thoracoscopic (VATS) procedure (112). In recent years, more complex intracardiac malformations have also been addressed by minimally invasive approaches. Septal defects, atrioventricular canal defect, valvular lesions (such as mitral cleft), anomalous pulmonic venous drainage, and even tetralogy of Fallot can be performed by limited right anterior thoracotomy, or lower partial sternotomy (110). These procedures, however, require modification of the cardiopulmonary bypass (CPB) management, including cannulation strategy and myocardial protection. Cannulation can be achieved peripherally *via* femoral and jugular cannulation, however, peripheral cannulation can be complicated and not feasible for children under 8 kg. The procedures can be performed either on a fibrillating heart or by cardioplegia infusion, depending on the type of the defect and the repair (113).

Future perspectives of minimally invasive techniques rely on endoscopic tools and robotic surgery. Nonetheless, contemporary existing instruments in the field are yet too big for neonatal thorax. The application of novel technology in the field will undoubtfully have a significant impact on the management of patient with congenital anomalies, and will affect the early and long term outcome of the repair (114).

#### Hybrid procedures in pediatric cardiac surgery

Tight collaboration between cardiac surgeons, interventional cardiologists and pediatric cardiac intensivists, has always been the hallmark of a well-functioning congenital cardiac center. Historically, however, this collaboration in the management of CHD has occurred in sequence. Hybrid approach for some of the congenital malformations consists of a combined interdisciplinary intervention in a single procedure (115). The goal of a hybrid procedure is to reduce the number of interventions and/or their invasiveness, decreasing by that the magnitude of cardiac interventions. Hybrid procedures are usually performed on a beating heart off CPB, which allows a real-time intraoperative feedback of a given procedure by angiography or by transesophageal echocardiogram (TEE).

Hybrid procedures are utilized under various circumstances. The classical indication for hybrid approach is for high risk, or low weight neonates with HLHS. Recent studies have demonstrated that these patients may benefit from a shorter and less invasive procedure, which consists of bilateral pulmonary artery banding and PDA stenting (116). The primary advantage of such procedures is in delaying major surgery with CPB in small neonates while improving hemodynamics to optimize growth and development despite possible risks of stent migration and the need for pulmonary artery reconstruction. Nonetheless, in spite of improvement in Norwood outcomes in the recent years, the hybrid approach is most commonly the procedure of choice in high risk or low birth weight neonates (<2 kg).

Muscular VSD is another indication for a hybrid approach. When surgery alone or catheter-based alone are unable to reach satisfactory results of a given defect, a hybrid approach may provide the solution. Muscular VSDs that are unreachable by surgery may be closed by a proper device on a non-heparinized heart *via* direct ventricular puncture using TEE guidance (117).

In summary, hybrid approach may be an excellent choice in selected patients. Advancements in this field are still required to provide better technical tool in order to reach improved outcomes, which will surely impact the complex management of these small patients.

### Mechanical circulatory support

Extracorporeal Life Support (ECLS) or Extracorporeal Membrane Oxygenation (ECMO) is a high-risk lifesaving advanced life support modality used in carefully selected patients with cardiorespiratory failure refractory to conventional therapeutic interventions. Despite ongoing evolution during the past 40 years, patient selection, minimizing ECMO/ECLS duration and complications, circuit pharmacology, and optimal anticoagulation remain some of the important challenges that ECMO/ECLS clinicians are aiming to overcome.

For cardiogenic shock, veno-arterial (V-A) ECMO can be utilized. Support is aimed at providing adequate systemic oxygen delivery, offloading the heart, and identifying/treating the underlying reason for cardiogenic shock as soon as possible. Future research should focus on optimizing patient selection and timing, single-ventricle support, outcome predictors, and the identification and treatment of residual lesions (118).

Extracorporeal cardiopulmonary resuscitation (ECPR) is the rapid deployment of V-A ECMO to provide cardiovascular support and gas exchange in the context of cardiopulmonary arrest, and can be considered for children with heart disease who experience a witnessed in-hospital cardiac arrest (119, 120). There is insufficient data to recommend ECPR for out-of-hospital cardiopulmonary arrest events in children. To further improve outcomes in ECPR, patient selection, team organization, high-quality CPR, measure and benchmark patient and process metrics, and simulation for individuals and team practice are key elements (119).

Utilization of ECMO for pediatric septic shock has not become a mainstay of sepsis protocols in most centers, but the surviving sepsis campaign guidelines do recommend to consider V-A ECMO as a rescue therapy in children with septic shock only if refractory to all other treatments (121), but there is still the need for more consistency in the indication criteria (122).

As the duration of ECMO/ECLS support and the occurrence of complications are important negative determinants of outcome, current and future endeavors at minimizing these factors are paramount. Systemic anticoagulation can be notoriously difficult, especially in infants, and the most common complications in all types of ECMO/ECLS support remain bleeding and/or thrombosis-related (123). In an attempt to minimize these often devastating complications, new ways of anticoagulating the circuit without anticoagulating the patient are being developed (124, 125). Surface modifications, aimed at overcoming the blood-biomaterial surface interactions, are currently being developed that mimic endothelium and anti-thrombotic agents (126). Three major groups of surface modifications are already in use or on the horizon. First, biomimetic surfaces such as heparin coating already exist, but do not obviate the need for systemic anticoagulation. Nitric-oxide donors from within the ECMO tubing, targeting platelet and fibrin adhesion as well as having antibacterial properties seem hopeful but are not yet commercially available. Secondly, biopassive surfaces such as phosphoryl or poly-2-methoxyethylacrylate coating have been shown to have a favorable effect on platelets by mimicking a biomembrane due to hydrophilic properties (127). The third surface modification aims to mimic endothelial function or to induce endothelialization of the actual surface itself. In the future a combination of biomimetic and bio-passive properties with a living cellular interface will likely become available (126). Until then, the most common systemic anticoagulant, heparin, remains the mainstay; however, the use of bivalirudin, a direct thrombin inhibitor, is becoming more prevalent (125, 128).

Pediatric ECMO/ECLS would benefit from smaller, safer, and smarter equipment, which would ideally act on feedback directly from patient parameters (e.g., temperature, blood pressure waveforms, continuous blood gas monitoring, etc.) to avoid hyperoxia, sudden drops in pCO2 and provide the ideal amount of flow. As far as we aware these interactive biofeedback ECMO systems are not under development (yet), but could surely play a role in the future. Other exciting innovations on the, more near, horizon are the use of pumpless ECMO and the development of an implantable artificial pediatric lung as a bridge to transplantation or lung remodeling for children with end-stage lung failure with promising results in animal models (129–131). Furthermore, development of an artificial placenta for premature infants also seems promising, but is not within the scope of this review (132). ECMO/ECLS is highly technical, requires expertise from many different specialties, and deserves rigorous initial and ongoing training (including simulation). The ECMOed taskforce from the Extracorporeal Life Support Organization (ELSO) has outlined an educational agenda with recommendations promoting an international collaborative approach toward standardization of ECMO education. High-quality research will be necessary to support educational practices (133).

Children who survive ECMO can suffer from a wide range of physical and neurodevelopmental disabilities, which they can even develop long after their stay in PCCC (134). Current data to support neuromonitoring on ECMO is limited. Therefore, future studies are needed to be able to develop evidence-based guidelines for neuromonitoring and neuroprotection for children supported with ECMO/ECLS (135). Moreover, very importantly, in the future, all ECMO/ECLS centers should have a structured long-term follow-up program to identify these disabilities early as recommended by ELSO (134).

Ventricular assist devices (VADs) are mechanical pumps that take over the function of the failing ventricle and restore adequate blood flow. Over the last few decades, significant effort has been dedicated to developing ventricular assist devices for smaller children with increasingly complex anatomy.

Short-term VADs are used in the acute treatment of cardiogenic shock or ventricular dysfunction after cardiac surgery with the expectation of patient recovery. These devices are deployed for hours to days as a “bridge to recovery” or “bridge to decision”. CentriMag (Thoratec Corporation, USA), and its pediatric version PediMag, are extracorporeal centrifugal pumps for short-term use as support for LV, RV or biventricular in children and adults. They have magnetically suspended rotors to minimize wear and the risk of hemolysis and thrombosis. Percutaneous devices such as Impella have been used successfully in bigger pediatric patients (more than 0.9 m^2^ of BSA), including patients with single ventricle physiology (136).

The Berlin Heart EXCOR (Berlin Heart GmbH, Berlin, Germany) is a pulsatile paracorporeal long-term device that can support patients in a wide range of sizes, from infants to teenagers as a bridge to cardiac transplantation in children with severe left or biventricular dysfunction. Even though not prospectively studied in children, intracorporeal adult VADs as Thoratec Heartmate II e III (Abbott corp, St. Paul, MN, USA), and Syncardia TAH (SynCardia Systems, Inc., Tucson, AZ, USA) can be used in older children and teenagers, with future perspectives for its usage as destination therapy.

VAD support for single ventricle physiology, especially after stage 1 and 2, is currently a challenge. However, it is possible to provide long term mechanical circulatory support for the Fontan population with end-stage heart failure to support the systemic circulation as a bridge to heart transplant (137). Moreover, the usage for long-term support for the pulmonary circulation in patients with univentricular physiology after Fontan procedure seems promising. Cysyk et al. reported a sheep study where a miniaturized device was successfully tested *in vivo* as a right heart replacement device demonstrating adequate circulatory support and normal physiologic pulmonary and venous pressures (138). Additional research is needed to continue to advance this promising approach.

### Safety and quality

PCCC has become incredibly complex due to patient heterogeneity and advances in medical and surgical strategies that have enabled treatment options for patients with increasingly complex conditions. An improved understanding of patient safety and quality of care and their impact on short and long-term outcomes is paramount to improve outcomes and decrease cost from complications (139, 140). As it is a fast-paced, technical environment with many distractions, complications and adverse events are frequently observed in the PCCC unit (141). High-risk procedures are being performed in complex patients with challenging physiology and anatomy with diverse teams. This requires high levels of technical and cognitive performance from staff. Hand offs, medication dispensing and administration, and diagnostic errors are a particular source of potential patient harm (142). Reducing risk of adverse events requires a safety culture which learns from previous incidents and proactively assesses risk of future events.

#### Errors, latent threats, culture and learning to improve safety

Both active errors and latent conditions impact patient safety in the PCCC unit. Active errors, those with an immediate detrimental effect, can either happen unconsciously or are deliberate violations of existing rules. Reducing these types of errors should be done at system level—analyzing and improving systems and processes to make it easier to accomplish high risk tasks in complex systems. For example, rules to limit distractions for medication preparation and mandatory double checks will reduce medication error. Latent conditions are factors that increase the likelihood of adverse incidents. Latent conditions known to the PCCC unit (143) include lack of crowd control during emergencies, lack of role clarity during surgical procedures, different structures to handover patients, inadequate equipment, shortage of staff, absence of senior staff, structural staff fatigue due to disproportionate workload, compassion fatigue and burn-out. Occupational stress is common in pediatric critical care and burn-out prevalence has been reported in ranges of 42–77% (144). Interventions to augment staff resilience such as education in self-care and peer support are indispensable for improvement of staff wellbeing, the perception of greater teamwork and ultimately patient safety and quality of care.

Organizational culture and team dynamics also play a major role in patient safety. Hierarchy remains a threat to patient safety (a nurse might find it difficult to address unsafe behavior of a doctor). Negative behaviors of healthcare staff are furthermore associated with decreased productivity, employee satisfaction, engagement and retention, increased absenteeism, poor teamwork and worse patient outcomes (145). Rude behavior within neonatal intensive care teams has been shown to negatively affect the ability of a team to diagnose and treat critically ill neonates (146).

There have been many reports of improvement of patient safety after systematic evaluation of safety threats (147). However, relying solely on incident reporting systems is insufficient to improve patient safety as a substantial number of incidents are not reported. Barriers to incident reporting including fear of retribution, inadequate reporting systems, lack perceived importance of reporting, lack of knowledge regarding safety event definitions, and lack of multidisciplinary collaboration in this process (148). Morbidity and mortality conferences and discussion of cases with excellent performance also positively impact outcome (149). The latter is associated with a more positive effect on healthcare staff than only learning from mistakes and near-misses and this may change the perception of reporting systems and increase overall reporting.

#### Information technology to enhance patient safety

There is an increased interest in the use of information technology and artificial intelligence to improve performance and overcome human error. Information technology has the potential to improve communication (i.e., handover summary), increase medication safety (i.e., notice of important medication interactions) and increase monitoring safety (i.e., alerting abnormal vital signs). Artificial intelligence is capable of analyzing and integrating the large volumes of continuous physiological data from patients in PCCC unit, predicting adverse events and ideally provide decision support as has been outlined in detail in the monitoring section.

#### Quality improvement in the PCCC unit by standardization of care and quality improvement programmes

Standardization is a way of dealing with human error by limiting options in the execution of care. Care can be standardized using guidelines, bundles, protocols and checklists. These tools improve adherence to best practice and contribute to patient outcomes. Bundles, protocols and pathways facilitate the development of shared expectations and understanding of standards of care for certain diagnoses and patients locally. The shared expectations set the framework for multi-disciplinary care delivery and teamwork. Examples are bundles and checklists to reduce adverse events during tracheal intubation, unplanned extubation, central line infections, cardiac arrest and the use of a handover checklist (150–152). Cognitive aid bundles for critical events improve adherence to best practice in simulation trials of these emergencies and their use is recommended (153).

An important issue for quality improvement in the care of children with congenital heart disease is the size of the case load of single centers. Failure to rescue (FTR)—the ability to prevent mortality following complications—is a potential challenge associated with a lower case volume and may represent some of the foundation to support centralization of care (154). But even single centers with higher case volumes may struggle to aggregate sufficient outcome data derive meaningful analyses in the short term (140). International societies and collaboratives such as the Extracorporeal Life Support Organization (ELSO), European Association for Cardio-Thoracic Surgery (EACTS), the Association for European Pediatric and Congenital Cardiology (AEPC) and the Cardiac Neurodevelopmental Outcome Collaborative have therefore developed guidelines to standardize care for children in the PCCC unit. The recent growth of quality improvement collaboratives and registries, facilitated by the evolution of videoconferencing is transforming quality and outcomes. These registries/collaboratives have been established with a focus on data sharing, commitment to high quality data and inclusion of multidisciplinary teams. Collaboratives such as the Pediatric Acute Care Cardiology Collaborative (PAC-3) (155), that achieved a reduction in length of postoperative hospital stay, the Pediatric Cardiac Critical Care Consortium (PC4), that achieved a 24, 22, and 12% relative reduction in in-hospital mortality, postoperative mortality and major complications, respectively (141). The National Pediatric Cardiology Quality Improvement Collaborative (NPC-QIC) successfully halved both mortality and growth failure in children with hypoplastic left heart syndrome between stage 1 and 2 palliation and demonstrate that there is a roadmap for multi-center collaborative quality improvement that results in sustained improvement in outcomes (156). Inclusion of developing countries in international collaboratives have also been successful (157) and increased international involvement must be a priority.

Congenital heart surgery outcome metrics have largely been defined in two domains, short and long-term mortality and short and long-term morbidity (158). As mortality rates have decreased over time, there is an increasing recognition of the importance of long-term neurodevelopmental and quality of life outcomes. Concurrently, there is growing recognition that survival in patients with complex, advanced illness may come at the cost of severe disability, negative quality of life of both patient and their family and increased healthcare costs (159). Benchmarking neurodevelopmental (intellectual, motor, developmental) and social outcomes for patients with CHD with the general population is an important tool to measure quality of care (160–162). There is a generally accepted multi-disciplinary set of long-term PICU outcome measures (163) and development of a similar set for patients in the PCCC unit should be considered.

The next steps to reduce mortality and make significant impact on long-term neurodevelopmental outcomes will require innovation and a keen focus on quality and safety. Learning from incidents, near-misses and excellence provides insight in ways to improve safety and care. Successful PCCC unit management highly depends on optimal multidisciplinary teamwork. Negative behaviors and other teamwork undermining factors should not be tolerated. Modern technology allows evaluation of practice through data sharing and machine learning algorithms and data integration remains an important next step in the advancement of our ability to utilize available data. Finally, long term outcome analysis should include considerations of means by which we can improve sustainability and cost of care delivery.

## Discussion

The future of PCCC appears bright with the array of emerging technologies. Investment in the human capital with advanced training and education, exploit artificial intelligence modalities into patient monitoring and early warning systems, personalized medicine with regenerative goals, improved surgical capabilities including minimally invasive as well as hybrid procedures, rescue therapy with cutting-edge mechanical circulatory support, and above all, the shield dome of patient safety and improved quality of care interact in harmony with each other to create the future stage of PCCC. The exponential convergence of these scientific and technological advances holds great promise to mitigate the disease burden of children with congenital heart disease.

## Author contributions

UP and DK contributed to conception, design, and integration of the manuscript. MEM and CM wrote the Education and training section. MM wrote the Patient monitoring section. YF wrote the Genomic medicine section. UP wrote the Regeneration, nanotechnology and tissue engineering section. EK and DM wrote the Alternative to traditional cardiac surgery. GL and PR wrote the Mechanical circulatory support section. DK and LK wrote the Safety and quality section. All authors contributed to manuscript revision, read, and approved the submitted version.

## Conflict of interest

The authors declare that the research was conducted in the absence of any commercial or financial relationships that could be construed as a potential conflict of interest.

## Publisher's note

All claims expressed in this article are solely those of the authors and do not necessarily represent those of their affiliated organizations, or those of the publisher, the editors and the reviewers. Any product that may be evaluated in this article, or claim that may be made by its manufacturer, is not guaranteed or endorsed by the publisher.
